# Supplementary figures and images for: Fast and versatile sequence-independent protein docking for nanomaterials design using RPXDock
Source: PLoS Comput Biol. 2023 May 22;19(5):e1010680. doi: 10.1371/journal.pcbi.1010680 (PMC10237659; doi:10.1371/journal.pcbi.1010680)

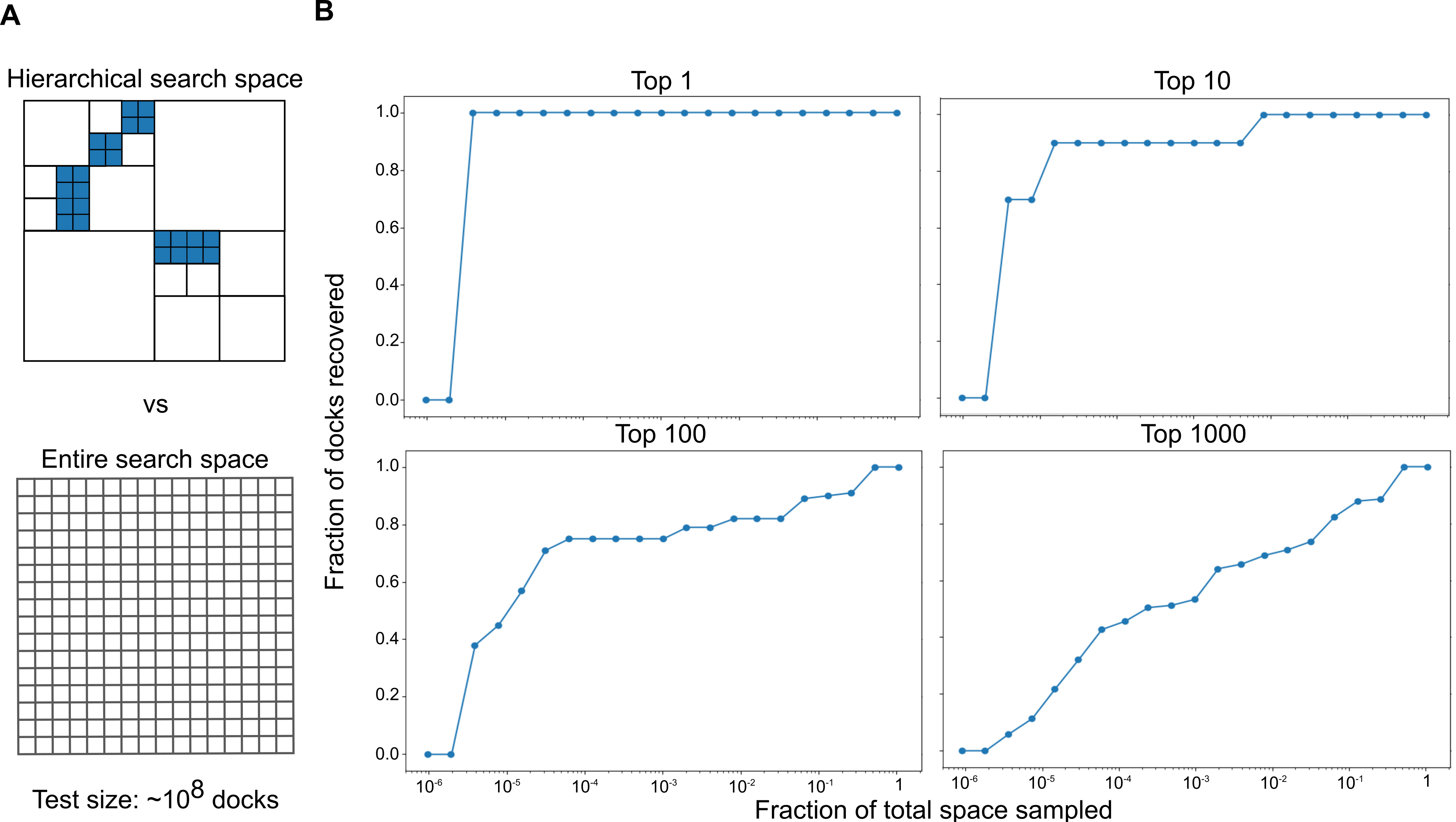

Supplement: S1 Fig — A. A 2-dimensional illustration of a hierarchical search grid with samples searched at the highest resolution in blue vs. a complete search grid at the same resolution. In this test dataset, ~108 total docks were sampled. B. A cumulative distribution of the fraction of the total search space that needs to be sampled in order to recover the top 1, 10, 100, and 1000 docks from this dataset. (TIF) [file pcbi.1010680.s001.tif]

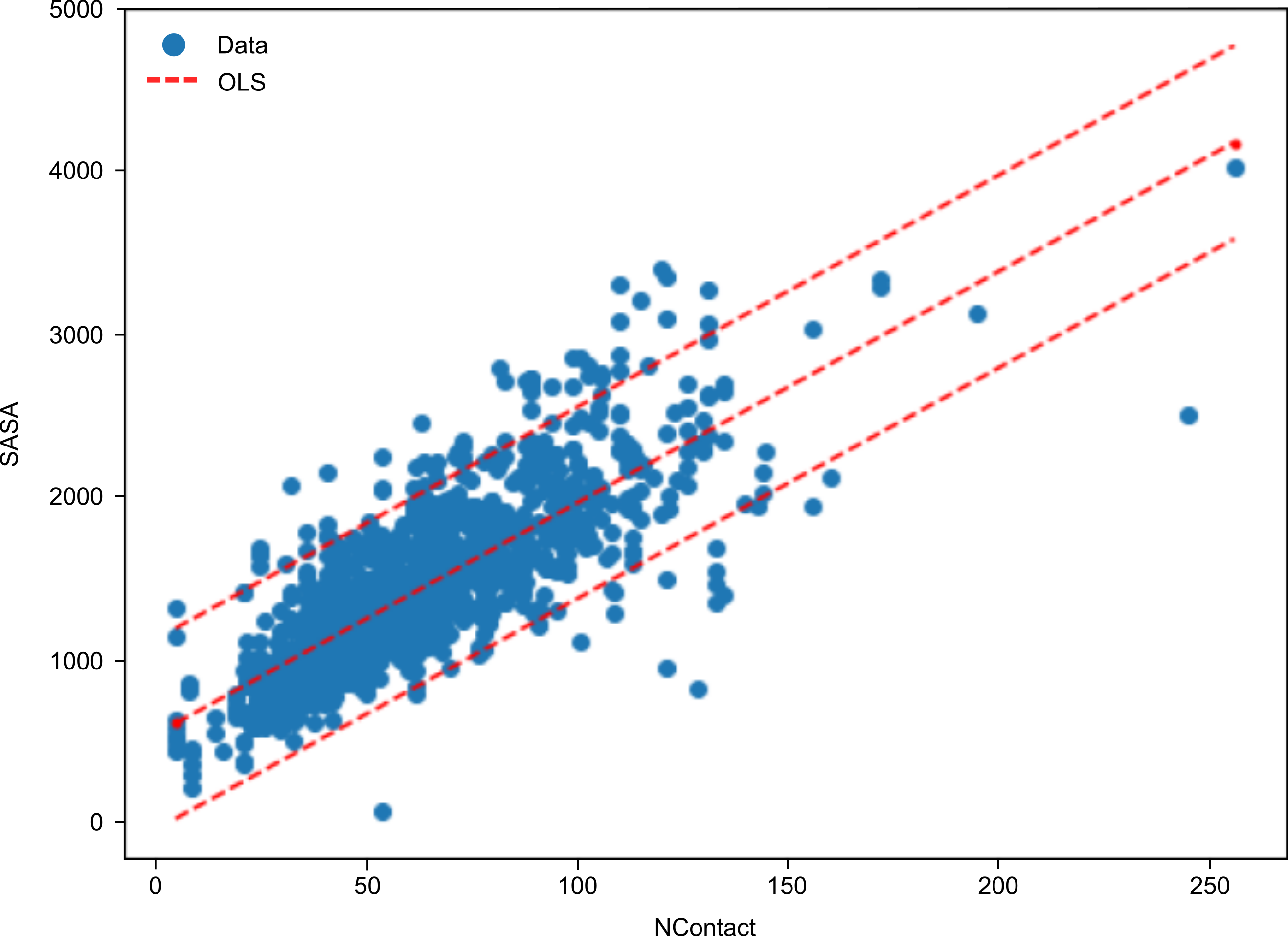

Supplement: S2 Fig — As such, we parameterized an ncontact score term with respect to computationally measured interface size, SASA. (TIF) [file pcbi.1010680.s002.tif]

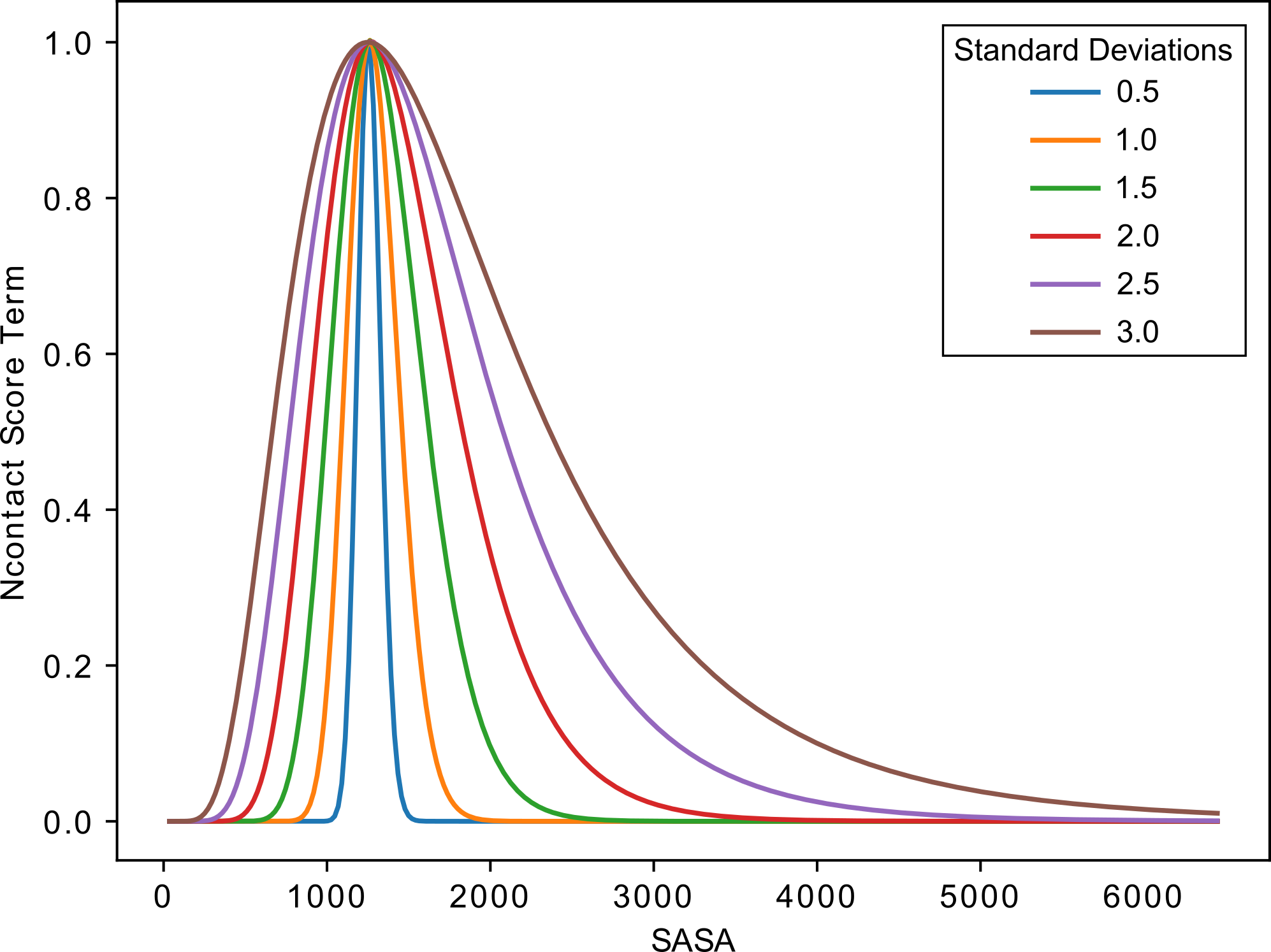

Supplement: S3 Fig — (TIF) [file pcbi.1010680.s003.tif]

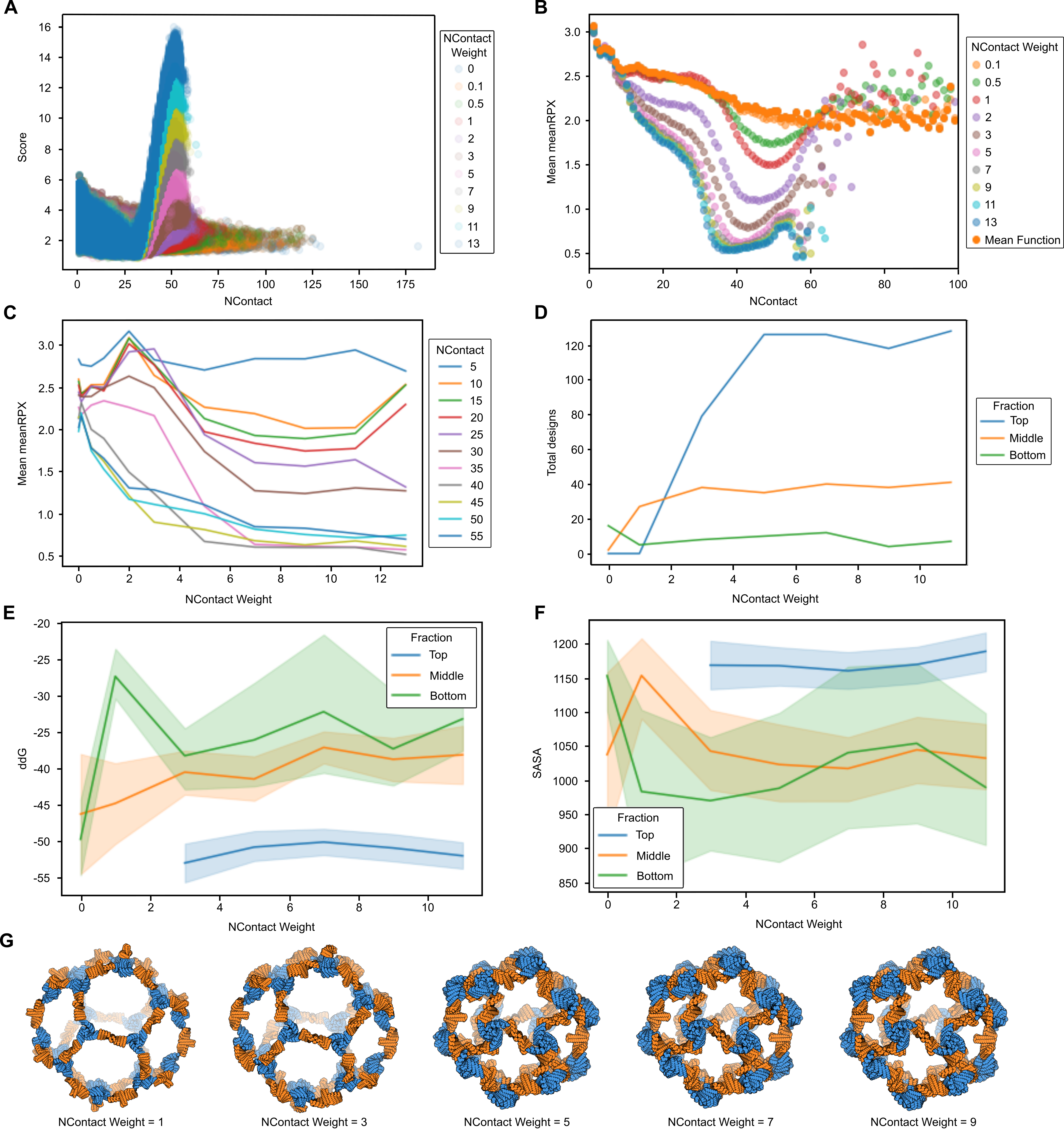

Supplement: S4 Fig — A. Score as a function of ncontact across various ncontact weights. B. Mean RPX as a function of ncontact. C. Mean RPX as a function of ncontact weighting plotted for interface sizes from Number of unique contacts = 5–55. D. Total number of passing designs out of 960 docks for each weighting and fraction. E-F. Computational design metrics as a function of ncontact weight for top-, middle-, and bottom-ranked designs for E. ddG, and F. SASA. G. The top dock with I32 icosahedral symmetry for, left to right, ncontact weight 1, 3, 5, 7, 9. (TIF) [file pcbi.1010680.s004.tif]

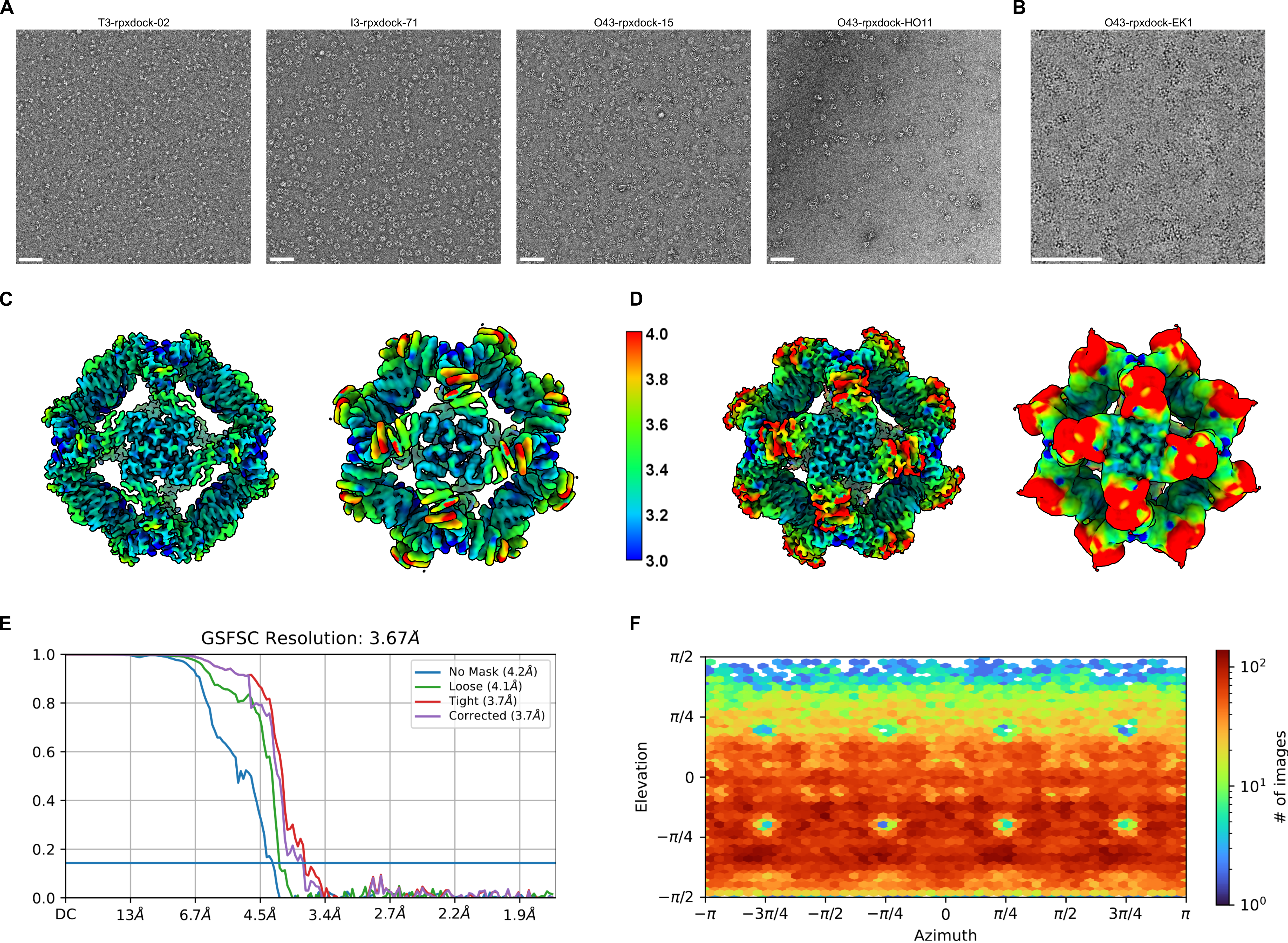

Supplement: S5 Fig — A. Representative raw nsEM micrographs of one- and two-component polyhedral self-assembling proteins from RPXDock. Scale bar = 100 nm B. Representative raw CryoEM micrograph showing good particle distribution and contrast of (Scale Bar = 100 nm). C. CryoEM local resolution map of O43-rpxdock-EK1, with the sharpened map at two different contour levels, using a tight mask, and calculated using an FSC value of 0.143. D. Local resolution estimates of the unsharpened map, also at two different contour levels (FSC = 0.143). The protruding arms of the designed cage only start to become visible at very low contour levels. Local resolution estimates range from ~3.2 Å at the core to >4.0 Å along the periphery of the extended arms due to a high degree of flexibility within this region. E. Global resolution estimation plot. F. Orientational distribution plot demonstrating near-complete angular sampling. (TIF) [file pcbi.1010680.s005.tif]
