# Supplementary material for: Fast and versatile sequence-independent protein docking for nanomaterials design using RPXDock
Source: PLoS Comput Biol. 2023 May 22;19(5):e1010680. doi: 10.1371/journal.pcbi.1010680 (PMC10237659; doi:10.1371/journal.pcbi.1010680)
Supplement: S1 Note — (DOCX) [file pcbi.1010680.s011.docx]

>T3-rpxdock-02

MGSVELLAVAALQELNIELARALLEAVARLQELNIDLVRKTSELTDEKTIREEIRKVKEESKRIVEEAEELIRLAKLASEAIARMAEVAARGAPPELLIELLERLLKKAQEAGMSPEIIHLLLELALAIVEARGVPPEQLAEFAERLVEILREAGGSPELVFELLKRIMEIIERRGAPPELLIELLERLLELAREAGLSPEQITKLLILALVIVMRRGVPPEQLAEFAEKLKEILREAGGSPELQRELKILIKLIEDLRGAGGSlehhhhhh

>I3-rpxdock-71

MKIEELFKKHKIVAVLRANSVEEAKKKALAVFLGGVHLIEITFTVPDADTVIKELSKLKEDGAIIGAGTVTSVEQCRKAVESGAEFIVSPHLDEEISQFCKEKGVFYMPGVMTPTELVKAMKLGHTILKLFPGEVVGPQFVKAMKGPFPNVKFVPTGGVNLDNVCEWFKAGVLAVGVGSALVKGEPVEVAEKAKAFVEKIRGCTElehhhhhh

>O43-rpxdock-15A

MGLEELLAKAAKDALSPDPEDLKEAVRLAEEVVRERPGSEAAKKALRIIQLAAELLKKSPDPEAIIAAARALLKIAATTGDNEAAKQAIEAASKAAQLAEQRGDDELVCEALALLIAAQVLLLKQQGVPMLEVAIHVAETILQILQRLKRKGASEEVRKECLKRILREIAEALQRSGVPEEEIALIMLLIILLLMML

>O43-rpxdock-15B

MGSVELLAVAALQELNIELARALLEAVARLQELNIDLVRKTSELTDEKTIREEIRKVKEESKRIVEEAEELIRLAKLASEAIARMAEVAARGAPPELLIELLERLLKKAQEAGMSPEIIHLLLELALAIVEARGVPPEQLAEFAERLVEILIRAGGSPELVFELLKRIMEIIERRGAPPELLIELLLNLLVLAVIAGLSPEQIHKLLEEALKIVERRGVPPEQLAEFAEQLKLILKLAGGSPELQKELKKEIEEIEQRRGAGGSGGSWGGlehhhhhh

>O43-rpxdock-HO11A

MSEELIREAVEAAKRFEEARKRFEEAKERGDEKEAREALKEMLRAIEELARVATELNDSRLVKAAAKLAIKLAEEALRFSDPEAAREAVRAALEIIRLMEKLAKKSNSEEIVELAARAAVELAAVAFQVGSSETARQAIETAARLIALLVELLKRRGTSEDEIAEIVARLISEIIRILKEANAEYKFICKAVAIVVAAIVEALKRSGTSEDEIAEIVARVISEVIRTLKESGSDYLIICVCVAIIVAEIVEALKRSGTSEDEIAEIVARVISEVIRTLKESGSSYEVIKECVQIIVLAIILALMKSGTEVEEILLILLRVKTEVRRTLKESGSWSG

>O43-rpxdock-HO11B

MLEMKLAVAELAAKSPDPELLKEAVKLAEEVVRERPGSEAAKKALEIIQEAAEKLKKSPDPEAIIAAARALLKIAATTGDNEAAKQAIEAASKAAQLAEQRGDDELVCEALALLIAAQVLLLKQQGVPMLEVAIHVAETILQILQRLKRKGASEEVRKECLKRILREIAEALQRSGVPEEEIALIMLLIILLLMMLGSWSGLEHHHHHH

>O43-rpxdock-EK1A

MALAYVMLGLLLSLLNRLSLAAEAYKKAIELDPNDALAWLLLGSVLEKLKRLDEAAEAYKKAIELKPNDASAWKELGKVLEKLGRLDEAAEAYLIAIMLDPEDAEAAKELGKVLEKLGELEMAEEAYKLAIKLDPND

>O43-rpxdock-EK1B

MEEAELAYLLGELAYKLGEYRIAIRAYRIALKRDPNNAEAWYNLGNAYTKQGDYDEAIEYYLRALVLDPNNAEAATNLGQAYMNQGDKDRAKLMLLLALKLDPNNDSARVILGVAKVGIEELAKLASQAQQEGDSEKQKAIELAAEAARVAQEVGDPELEKLALEAARRGDSEKAKAILLAAEAARVAKEVGDPELIKLALEAARRGDSEKARAILEAAERAREAKERGDPEQIKKARELAKRLEHHHHHH
