# Supplementary material for: Fast and versatile sequence-independent protein docking for nanomaterials design using RPXDock
Source: PLoS Comput Biol. 2023 May 22;19(5):e1010680. doi: 10.1371/journal.pcbi.1010680 (PMC10237659; doi:10.1371/journal.pcbi.1010680)
Supplement: S1 Text — (DOCX) [file pcbi.1010680.s012.docx]

# Supplemental Information

## Bodies

The body class uses pyrosetta to access the pose and initial coordinates of the input .pdb files for a particular docking trajectory. From the pyrosetta pose, the body class stores chain, sequence, secondary structure, and backbone positional information of the asymmetric unit. In this class, any user inputs to allow only certain portions of the pose for docking are also stored (--allowed_residues, --term_access. Only the initial coordinates of the pyrosetta pose are stored in the body class, while the transformation matrix generated by the search is applied to the initial coordinates of the starting pose. Backbone positional information derived from the transforms are stored as clouds of points during the hierarchical search. The body class checks for clashes between transformed backbones by looking for intersections between these clouds of points at each level of the bounding volume (intersect_range, intersect, and clash_ok). At lower resolutions, the clouds of points are smoother and larger, and at higher resolutions, the clouds of points are smaller. Pairs of contacting positions and secondary structure elements (contact_pairs) and counts of contacting pairs (contact_count) are also evaluated in the body class.

## Search

The search module contains the core code controlling the search process. It contains the two fundamental search methods, hierarchical and grid, the geometric specifications (*spec*) for each architecture, the allowed degrees of freedom (*sampler*), and a module for each docking application depending on the architecture and number of bodies. Which module is used depends on the specific architecture and is called by dock.py. They are *asym*, *cyclic*, *onecomp*, and *multicomp*. There are also the special-use search applications for one-dimensional (*helix*), and stacking (*axle*) architectures. Finally, the *search* module contains a *result* object which defines the *result* class and associated functions.

Each module type (*asym*, *cyclic*, *onecomp*, *multicomp,* etc.) has a *make function*, e.g., make_multicomp() and one or more *evaluator functions*. The *make function* takes as required arguments a body or bodies, a spec, a motif-score hash-table (hscore), a search method (default hier_search), and a sampler (default None). The sampler is hier_multi_axis_sampler() for multicomp and hier_axis_sampler() for onecomp. *Make functions* manage the execution of the docking process by setting up the evaluator function, which performs a redundancy check and calls any specified filters. The evaluator function, in conjunction with the search method, will evaluate the transforms and scores from the sampler at each search resolution and return the top-scoring transforms to be expanded in the next level of search resolution, affecting the docking trajectory. Finally, at the end of docking, the *evaluator function* generates and returns a result object. Result objects are described in further detail in the main text.

## Score

The sasa_priority score function takes into consideration both the quality of the motifs in the interface, and also how far the interface is from a desired size. To develop this function, we generated a predictive model by docking a set of oligomeric scaffolds in all two-component polyhedral group architectures using the stnd score function and designed the novel protein-protein interfaces of a random selection of docks using the Rosetta software suite to obtain buried SASA scores [[1]](https://paperpile.com/c/6lvXgf/1M7F8). The resulting model fit to the relationship is $SASA = 29.1*ncontact + 282, R^{2}=0.634$. Because ncontact correlates strongly with the computationally measured interface size, SASA, we parameterized an ncontact score term with respect to SASA over a range of plausible interface sizes and standard deviations (**S2 Fig**).

We fit the correlation between the distribution mean and mode of computationally predicted SASA with a linear regression, and the relationship between the standard deviation and the slope of the correlation between the mean and mode of the distribution with a Gaussian decay function. The resulting log-normal distributions have a maximum score at the input SASA, and the score is invariant with respect to the standard deviation (**S3 Fig**).

The final score function contains an *RPX* score term and an ncontact score term: $score = a*\underline{X}_{RPX} + b*ln N\left( \mu,\sigma^{2} \right)$. The *RPX* score term consists of a scalar multiplier of $\underline{X}_{RPX}$ , which refers to the average of the best motifs found across all residue pairs and is used as an approximation of interface quality (Default 1.0). The ncontact score term scores the number of unique contacting pairs (N) based on a log-normal distribution set by μ, the desired SASA set by the user, and σ, a user-defined tolerance value that is a scalar multiplier of the standard deviation of the fit error for the correlation between SASA and ncontact, i.e., the accuracy of the prediction for the desired SASA (default 4). Since the *RPX* score term tends to bias towards interfaces that have few but very high quality motif pairs, the weight for the ncontact score term, *b*, needs to be scaled appropriately to overcome the $\underline{X}_{RPX}$ tendency towards small interfaces.

To determine the appropriate default value for *b*, we systematically varied it from 0 to 13 and docked a standard set of scaffolds. The top-, middle-, and bottom-ranked docks were designed using tools/cage_design.xml, included in the GitHub repository. Increasing the value of *b* made the interface-size bias to the total score more pronounced, but somewhat surprisingly decreased the maximum interface size observed in all docks (**S4A Fig**). The weighting also had an unexpected effect on the average RPX score: The $\underline{X}_{RPX}$ also decreased around the desired SASA as *b* increased (**S4B Fig**). Because $\underline{X}_{RPX}$ is calculated using the mean() gather function, as opposed to a sum() as in the stnd score function, this result can be interpreted as the ncontact score term weight having a negative impact on the interface quality for a given interface size. This effect converges above an ncontact weighting of 5, although convergence depends on user-defined interface size (**S4C Fig**).

We also evaluated the 960 top-, middle-, and bottom-scoring docks for each weighting of the ncontact score term and a target SASA of 1125 Å^2^ against Rosetta design computational filters including ddG < -20 and SASA between 850 Å^2^ and 1200 Å^2^ (included in cage_design.xml). Despite the apparent decrease in interface quality as a function of increasing ncontact weight, weights of 5 and above resulted in a higher percentage of top-scoring docks passing Rosetta design filters (**S4D Fig**). There was also almost no difference in computationally estimated ddG or SASA for top docks above an ncontact weight of 3 (**S4E-F Fig**). In fact, no statistically significant difference between weights could be detected for any computational design metric. Qualitatively the designs from each weighting look similar, with the top dock for each weight converging after weight = 5 (**S4G Fig**). Therefore, we selected a default ncontact weight of 5 as the most conservative weighting that also maximizes the design success rate.

## Filters

The filter module serves two purposes. The first is to filter redundant docks during the search process, described in the Clustering section. The second is to use the filter() function to execute an arbitrary number of filters, defined by the user in a filter config file (in .yaml format). This function takes in the body object and transforms, and parses the config file set with the --filter_config argument, calling any filters defined in the config file. For all filters, the filter() function returns an array of indices for docks passing all filters if the “confidence” configuration is set to *True*. The function also returns any extra data provided by the filters. Available filters are filter_sasa() and filter_sscount().

**The SASA filter** attempts to estimate the Solvent Accessible Surface Area buried by the formation of a protein-protein interface. The estimate is based on a linear fit of SASA, calculated by the SASA filter in Rosetta, as a function of the number of unique residues in a docked interface. The filter_sasa() function takes as arguments transforms and bodies, as well as parsed keyword arguments from the config file. A full list of options for the SASA filter can be found in **Table S2**.
**The sscount filter** attempts to estimate the number of secondary elements in contact at a protein-protein interface. The filter uses the secondary_structure_map class, which maps secondary structure elements (either Helix, Sheet, or Loop), based on user-definitions of each secondary structure element, onto the body object. Secondary structure elements are recorded for a particular body object if a consecutive stretch of identified secondary structure types exceed a given minimum residue length controlled by min_helix_length, min_sheet_length, and min_loop_length. Given the number of unique pairs of residue contacts at a protein-protein interface, the filter_sscount()function estimates the number of each secondary structure element type that is in contact at the interface. A full list of options for the sscount filter can be found in **Table S3**.

## Experimental Material and Methods

### Computational design

As inputs to RPXDock we used one native scaffold (PDB ID: 1wa3) and cyclic oligomers of either C3 or C4 symmetry generated via rigid helical fusion from validated oligomeric scaffolds and de novo helical repeat proteins as input building blocks for RPXDock [[2–5]](https://paperpile.com/c/6lvXgf/W4rZ5+iRNre+EeLRs+npF3j). Table S4 describes the input pdb files used to generate each dock, and the asymmetric units of each input pdb file are provided in the tools/inputs/scaffolds/ directory of the RPXDock GitHub page (<https://github.com/willsheffler/rpxdock>). Docks were generated using the tools/dock.sh file, also provided on GitHub. We used the sasa_priority score function, providing a value of 1500 or 1125 for the --weight_sasa option for one- and two-component docking problems, respectively. Docks were allowed to sample a Cartesian bound space between 0 and 300 Å with the ailv_h motif settings. The sequences of the interfaces for the top 10 docks for each scaffold (one-component) or scaffold pair (two-component) were optimized symmetrically using Rosetta sequence design (Leman et al. 2020) with the tools/rpxdock_to_design.xml file provided on GitHub. Designable residues at the docked interfaces were selected using Rosetta-based interface selection task operations. The designable residues were split into core, boundary, and surface layers with residue selectors and designed via layer design followed by side chain minimization [[1,6]](https://paperpile.com/c/6lvXgf/1M7F8+krt8C). The number of side-chain dependent clashes, interface size, and the predicted binding energy of the complexes (ddG) were then calculated for each sequence using Rosetta-based filters.

### Protein expression and purification

Synthetic genes were optimized for *E. coli* expression and purchased from IDT (Integrated DNA Technologies) as plasmids in the pET29b expression vector encoding a C-terminal hexahistidine affinity tag. Bicistronic genes encoding two components were joined together by a short intergenic region containing a ribosome binding site (gene sequence: TAAAGAAGGAGATATCATATG) and the hexahistidine tag was included on only one of the two components. Expression plasmids were transformed into BL21(DE3) *E. coli* competent cells (Invitrogen). Single colonies from agar plate with 100 mg/L kanamycin were inoculated in 50 mL of Studier autoinduction media (Studier and William Studier 2005), and the expression continued at 37 °C for over 24 hours. Cells were harvested by centrifugation at 4000 g for 10 min, and resuspended in 2.66 - 35 mL lysis buffer of 300 mM NaCl, 25 mM Tris pH 8.0, 1 mM PMSF, 0.25 mg/mL lysozyme, and 0.25 mg/mL DNase. After lysis by sonication and centrifugation at 14000 g for 45 min, the supernatant was purified by Ni^2+^ immobilized metal affinity chromatography (IMAC) with Ni-NTA Superflow resin (Qiagen). Resins with bound cell lysate were washed with 10 mL (bed volume 1 mL) of washing buffer (300 mM NaCl, 25 mM Tris pH 8.0, 60 mM imidazole) and eluted with 5 mL of elution buffer (300 mM NaCl, 25 mM Tris pH 8.0, 300 mM imidazole). Concentrated samples were purified by SEC in 300 mM NaCl, 25 mM Tris pH 8.0 on a Superose 6 Increase 10/300 gel filtration column (Cytiva).

### Negative-Stain Electron Microscopy (nsEM)

Assemblies were diluted to ~0.1 mg/mL (asymmetric unit concentration) and applied onto glow discharged, carbon supported 300-mesh copper grids (Ted Pella, Inc.), followed by 2× application of 4 μl 2% uranyl formate stain. Micrographs were recorded using EPU software (Thermo Fisher) on a 120 kV Talos L120C transmission electron microscope (Thermo Scientific) at a pixel size of 2.47 Å per pixel and a defocus range of 1.0 to 2.5 μm.

### Negative Stain Electron Microscopy image processing

nsEM datasets were processed by CryoSPARC software v4.0.3 (Punjani et al. 2017). Micrographs were imported into the CryoSparc software. Around 200 particles were manually picked, 2D classified and selected classes were used as templates for particle picking in all images. All the picked particles were 2D classified for 40 iterations into 50 classes. Particles from selected classes were used for building three asymmetric ab-initio initial models. Each model was then homogeneously refined using C1 and the corresponding T/O/I symmetry independently.

### CryoEM sample preparation and data collection

3 µl of O43-EK1 sample at 0.8 mg/mL in 25 mM Tris pH 8.0, 150 mM NaCl was applied onto C-flat 1.2/1.3 holey carbon grids overlaid with a thin layer of continuous carbon. Grids were then plunge-frozen into liquid ethane and cooled with liquid nitrogen using a ThermoFisher Vitrobot Mk IV with 0.5 s blotting time, a 5 second wait time, and 0 blot force. The blotting process took place inside the vitrobot chamber at 22°C and 100% humidity. Data acquisition was performed with SerialEM on a ThermoFisher Glacios electron microscope operating at 200 kV using a K3 Summit direct electron detector operating in super-resolution mode. The nominal magnification for data collection was 45000× with a calculated pixel size of 0.4425 Å/pixel, with a final dose of 50 e-/A2 for 1638 movies.

### CryoEM data processing

The raw micrographs were collected on the ThermoFisher Glacios electron microscope using SerialEM and were processed in CryoSPARC v3.0.0, v4.0.2, and v4.0.3 [[7]](https://paperpile.com/c/6lvXgf/Tdw9N). The 1638 raw movies with a raw pixel size of 0.4425Å, total exposure dose of 50 e/Å^2^, and spherical aberration of 2.7 mm at an accelerating voltage of 200 kV were imported into the CryoSPARC software package. The imported micrographs were motion corrected using Patch Motion Correction with the maximum alignment resolution of 5, F-crop output factor of 0.5, and B-factor of 500. The motion corrected micrographs were CTF corrected using Patch CTF with amplitude contrast of 0.1, a resolution range between 4 and 21 Å, search defocus range between 1000 and 40000 Å, and search phase shift range between 0 and π radians. 4000 peaks per micrograph were chosen using Blob Picker, with particle diameter set between 175 and 325 Å and a low pass filter of 20 Å applied to both templates and micrographs. Particle picks were manually curated to exclude ice and noise by adjusting NCC and local power thresholds using Inspect Picks. Selected particles were extracted at a box size of 564 pixels and F-cropped to 282 pixels. Particles were sorted into 100 2D class averages over 40 iterations of classification with a batch size of 200. 2D classes showing assembled cages and clear secondary structural features were selected as templates for a second round of particle picking. The template picked particles were iteratively sorted by 2D classifications using varying parameters, and the selected particles were used to generate a single 3D *ab initio* volume using imposed octahedral symmetry, with minimum and maximum resolutions of 34 Å and 12 Å, respectively. The 3D *ab initio* volume was used as the initial reference for a non-uniform refinement that reached a GSFC of 3.73 Å. The unsharpened volume generated from the non-uniform refinement was used to create a mask around the rigid region of the cage, excluding the flexible arms, lowpass filtered by 15 Å, with a dilation radius of 3 Å and soft padding of 5 Å. The selected particles were re-extracted at a box size of 380 pixels, without F-cropping, and were run through Exposure Group Utilities, splitting the input particles into smaller subsets using a blob/path result field, string_spl token creation strategy, split group index of 5, and fail combine strategy. The Exposure Group Utilities output particles were used to create a non-uniform refinement with imposed octahedral symmetry, initial lowpass resolution of 12 Å, and GSFSC split resolution of 20 with optimized per-particle defocus and optimized per-group CTF parameters. The output volume and particles were used with the previously generated mask to perform a local refinement with window inner and outer radii of 0.65 and 0.7, 20 degree rotation search extent, 10 Å shift search extent, 0.2 degree maximum alignment resolution, imposed octahedral symmetry, 12 Å initial lowpass resolution, non-uniform filter order 2, batchsize epsilon of 0.001, batch size snr factor of 50, and a 34 Å GSFSC split resolution using a symmetric noise model and noise initial sigma scale of 3. The local refinement yielded a final 3.67 Å map. For comparison, an identical local refinement was performed without any symmetry imposed on the particles at any stage, resulting in a 5.37 Å map.

### CryoEM model building and validation

A Gaussian version of the unsharpened local refinement map was created in UCSF ChimeraX [[8]](https://paperpile.com/c/6lvXgf/gsH8N) and the design model was iteratively relaxed into the Gaussian, unsharpened, and sharpened versions of the map. Each iteration used the previous output structure as the input structure for the density guided relaxation tool, Namdinator [[9]](https://paperpile.com/c/6lvXgf/pdmIi). The final Namdinator output model was aligned with the sharpened map. ISOLDE [[10]](https://paperpile.com/c/6lvXgf/IwpRV) was used to run a global simulation of molecular dynamics based on the AMBER force field model, followed by smaller simulations on each chain individually and the regions containing interfaces and by local simulations and manual adjustments of Ramachandran outliers, rotamer outliers, and steric clashes above 0.5 Ångstroms, alternating with periodic global simulations to avoid local minima. The manually adjusted ISOLDE output model was real space refined in Phenix to correct for unfavorable bond lengths and angles. The remaining Ramachandran outliers, rotamer outliers, and clashes were conservatively adjusted in ISOLDE before a single global minimization macrocycle in Phenix. Finally, side chains and sections of the backbone that were not clearly supported by density were deleted from the final solved structure and the final structure was evaluated in Phenix, showing a clash score of 1.5, molprobity score of 0.89, 0% Ramachandran outliers, 0% CBeta outliers, 0.05% rotamer outliers, 0% twisted prolines, 0.14% CaBLAM outliers, bond length RMSD of 0.003 Å, bond angle RMSD of 0.522 (0) degrees, and whole, helix, and loop Ramachandran plot Z scores (RMSDs) of 1.38 (.11), 1.01 (0.07), and 0.94 (0.32), respectively. The final coordinates and cryoEM maps for O43-rpxdoc-EK1 were deposited in the Protein Data Bank and Electron Microscopy Data Bank under accession numbers PDB: 8FWD and EMD-29502, respectively.

####

#

# References

1. [Bale JB, Gonen S, Liu Y, Sheffler W, Ellis D, Thomas C, et al. Accurate design of megadalton-scale two-component icosahedral protein complexes. Science. 2016 Jul 22;353(6297):389–94.](http://paperpile.com/b/6lvXgf/1M7F8)

2. [Fallas JA, Ueda G, Sheffler W, Nguyen V, McNamara DE, Sankaran B, et al. Computational design of self-assembling cyclic protein homo-oligomers. Nat Chem. 2017 Apr;9(4):353–60.](http://paperpile.com/b/6lvXgf/W4rZ5)

3. [Brunette TJ, Parmeggiani F, Huang PS, Bhabha G, Ekiert DC, Tsutakawa SE, et al. Exploring the repeat protein universe through computational protein design. Nature. 2015 Dec 24;528(7583):580–4.](http://paperpile.com/b/6lvXgf/iRNre)

4. [Hsia Y, Mout R, Sheffler W, Edman NI, Vulovic I, Park YJ, et al. Design of multi-scale protein complexes by hierarchical building block fusion. Nat Commun. 2021 Apr 16;12(1):2294.](http://paperpile.com/b/6lvXgf/EeLRs)

5. [Boyken SE, Chen Z, Groves B, Langan RA, Oberdorfer G, Ford A, et al. De novo design of protein homo-oligomers with modular hydrogen-bond network-mediated specificity. Science. 2016 May 6;352(6286):680–7.](http://paperpile.com/b/6lvXgf/npF3j)

6. [Hsia Y, Bale JB, Gonen S, Shi D, Sheffler W, Fong KK, et al. Design of a hyperstable 60-subunit protein icosahedron. Nature [Internet]. 2016; Available from:](http://paperpile.com/b/6lvXgf/krt8C) <http://dx.doi.org/10.1038/nature18010>

7. [Punjani A, Rubinstein JL, Fleet DJ, Brubaker MA. cryoSPARC: algorithms for rapid unsupervised cryo-EM structure determination. Nat Methods. 2017 Mar;14(3):290–6.](http://paperpile.com/b/6lvXgf/Tdw9N)

8. [Pettersen EF, Goddard TD, Huang CC, Meng EC, Couch GS, Croll TI, et al. UCSF ChimeraX: Structure visualization for researchers, educators, and developers. Protein Sci. 2021 Jan;30(1):70–82.](http://paperpile.com/b/6lvXgf/gsH8N)

9. [Kidmose RT, Juhl J, Nissen P, Boesen T, Karlsen JL, Pedersen BP. Namdinator - automatic molecular dynamics flexible fitting of structural models into cryo-EM and crystallography experimental maps. IUCrJ. 2019 Jul 1;6(Pt 4):526–31.](http://paperpile.com/b/6lvXgf/pdmIi)

10. [Croll TI. ISOLDE: a physically realistic environment for model building into low-resolution electron-density maps. Acta Crystallogr D Struct Biol. 2018 Jun 1;74(Pt 6):519–30.](http://paperpile.com/b/6lvXgf/IwpRV)
