# Supplementary material for: Fast and versatile sequence-independent protein docking for nanomaterials design using RPXDock
Source: PLoS Comput Biol. 2023 May 22;19(5):e1010680. doi: 10.1371/journal.pcbi.1010680 (PMC10237659; doi:10.1371/journal.pcbi.1010680)
Supplement: Attachment — Submitted filename: RPXDock_ms_response_to_reviewers.pdf [file pcbi.1010680.s013.pdf]

Reviewer 1

The paper describes a new computational procedure for docking proteins into user-defined oligomeric symmetries. A number of techniques addressing this problem have been developed by others. However, the procedure described in the paper emphasizes flexibility of its application to a variety of tasks, and utility for the practical use in protein design, including completeness of user's documentation, accessibility to the users community, etc. The procedure is properly benchmarked and described in great detail. As such it should be a useful addition to the toolchest of protein design community.

We thank the reviewer for their positive assessment of our work.

A small technical glitch - Supplemental Figure S1 is placed in the main text, instead of the Supplement.

This is a great catch and we have updated our supplemental information to contain a supplemental figures section where we've moved Figure S1.

Reviewer 2

This paper is the description of RPXDock, a software package for sequence-independent rigid-body protein docking across a wide range of symmetric architectures, suitable for large constructs of 1 or 2 types of proteins towards desired configurations. The software is novel and potentially very useful.

We thank the reviewer for their positive assessment of our work.

However, I consider it a problem that while the description of the methods and use options is complete and very detailed, there is not a single example demonstrating the use of the software. I think this is a missing component. The paper states that "RPXDock was used to successfully design cyclic oligomers (Gerben et al., submitted), one-component nanocages (Wang et al. 2022), two-component nanocages (Li et al., submitted; Huddy et al., in preparation; Dosey et al., in preparation), and even larger pseudo-symmetric nanomaterials (Dowling et al., in preparation; Lee et al., in preparation), establishing its utility and generality." Thus, the only paper currently available is Wang et al. "Improving the Secretion of Designed Protein Assemblies through Negative Design of Cryptic Transmembrane Domains." in preprint, and it focuses on the analysis of the designed assemblies rather than on their design. All the other paper are submitted or in preparation, and do not demonstrate the methodology. Thus, the performance of the method can be fully understood only by downloading and installing the software, which makes it difficult to form an informed opinion. I understand that the authors may want to publish the methodology prior to application papers, but I think including some demonstration of results would make this paper more interesting to readers.

We thank the reviewer for this suggestion. We've added a section and figure in the main text to address this:

"Experimental characterization of one- and two-component polyhedral self-assembling proteins from RPXDock

We set out to experimentally evaluate symmetric one- and two-component structures with polyhedral group symmetry generated using RPXDock. Given a set of prevalidated homomeric scaffolds with cyclic symmetry, we generated docks using RPXDock, and the resulting interfaces were sequence-optimized via Rosetta or ProteinMPNN sequence design (Leman et al. 2020; Dauparas et al. 2022). Two one-component designs (T3-rpxdock-02, I3-rpxdock-71) and two two-component designs (O43-rpxdock-15, O43-rpxdock-HO11) with tetrahedral, octahedral, or icosahedral symmetry were examined by negative-stain electron microscopy and found to adopt the intended architecture (Fig 5A-D, Fig S5A). I3-rpxdock-71, while completely independently sampled and designed, resembles a dock previously sampled by RPXDock's predecessor, tcdock, indicating that the similar top results are identified by the new search algorithm (Hsia et al. 2016). We obtained a 3.7 Å resolution single-particle reconstruction of the two-component octahedral assembly O43-rpxdock-EK1 (PDB: 8FWD, EMD-29502) using cryogenic electron microscopy and found that it assembles to the intended structure with high accuracy (4 Å Cα root mean square deviation between all 48 chains of the original dock and cryoEM structure; Fig 5E, Fig S5B-F, Table S5). Together, these data confirm that docks generated using RPXDock can be designed to assemble in the intended configurations without disrupting the integrity of the starting scaffolds. Design models are available as a supplemental file."

We believe that the variety of symmetric architectures we characterize experimentally, including both one- and two-component assemblies, demonstrate the performance of the method. We have also included supplementary methods and data corresponding to the experiments we performed to validate the RPXDock method.
