# Supplementary material for: Fast and versatile sequence-independent protein docking for nanomaterials design using RPXDock
Source: PLoS Comput Biol. 2023 May 22;19(5):e1010680. doi: 10.1371/journal.pcbi.1010680 (PMC10237659; doi:10.1371/journal.pcbi.1010680)
Supplement: Attachment — Submitted filename: 20230212 RPXDock_ms_response_to_reviewers.pdf [file pcbi.1010680.s014.pdf]

The experimental validation provided is extremely impressive and illustrate well the functionality of the algorithm. However, more detail needs to be provided for how the experimental validation was preformed for usability/reproducibility. Please include details of:

1) what proteins were used

2) which parts of the protein were redesigned using Rosetta or ProteinMPNN

3) which of those two methods was used for each protein

and 4) the parameters that were used in each case.

We thank the editors for this suggestion. We have provided input pdb files, all of the scripts used for docking and sequence design, and descriptions of each script in an accompanying README document in our RPXDock GitHub repository at https://github.com/willsheffler/rpxdock. We've also added the following section in the supplemental information to provide the requested details to the reader:

Computational design

As inputs to RPXDock we used one native scaffold (PDB ID: 1wa3) and cyclic oligomers of either C3 or C4 symmetry generated via rigid helical fusion from validated oligomeric scaffolds and de novo helical repeat proteins as input building blocks for RPXDock (Fallas et al. 2017; Brunette et al. 2015; Hsia et al. 2021; Boyken et al. 2016, Huddy et al. in preparation, Edman et al. submitted). Table S4 describes the input pdb files used to generate each dock, and the asymmetric units of each input pdb file are provided in the inputs/ directory of the RPXDock GitHub page (https://github.com/willsheffler/rpxdock). Docks were generated using the tools/dock.sh file, also provided on GitHub. We used the sasa_priority score function, providing a value of 1500 or 1125 for the --weight_sasa option for one- and two-component docking problems, respectively. Docks were allowed to sample a Cartesian bound space between 0 and 300 Å with the ailv_h motif settings. The sequences of the interfaces for the top 10 docks for each scaffold (one-component) or scaffold pair (two-component) were optimized symmetrically using Rosetta sequence design (Leman et al. 2020) with the tools/rpxdock_to_design.xml file provided on GitHub. Designable residues at the docked interfaces were selected using Rosetta-based interface selection task operations. The designable residues were split into core, boundary, and surface layers with residue selectors and designed via layer design followed by side chain minimization (Bale et al. 2016). The number of side-chain dependent clashes, interface size, and the predicted binding energy of the complexes (ddG) were then calculated for each sequence using Rosetta-based filters.

Finally, we updated Table S4 to indicate the input pdb file that was used for each input argument in the RPXDock program to generate the novel nanomaterials:

Table S4: Design construct renaming and input pdb files

Published name	Original name	Inputs1	Inputs2
T3-rpxdock-02	cage_twtls-02	C3_hfuse_twtls_003_4x_asu.pdb	
I3-rpxdock-71	I3-71_M3I	C3_1wa3_asu.pdb	
O43-rpxdock-15	cage_twtls-15	C4_171-7_asu.pdb	C3_hfuse_twtls_003_4x_asu.pdb
O43-rpxdock-HO11	O43-HO11	C4_171-7_asu.pdb	C3_HO10_asu.pdb
O43-rpxdock-EK1	O43-EK1	C4_tpr1C4-pm3_asu.pdb	C3_1na0HFuse_015_asu.pdb
